# The multifaceted roles of natural products in mitochondrial dysfunction

**DOI:** 10.3389/fphar.2023.1093038

**Published:** 2023-02-13

**Authors:** Qianrun Chen, Danhua Ruan, Jiayan Shi, Dongru Du, Ce Bian

**Affiliations:** Key Laboratory of Birth Defects and Related Diseases of Women and Children , Department of Gynecology and Obstetrics, Ministry of Education, West China Second Hospital, Sichuan University, Chengdu, China

**Keywords:** natural products, mitochondria, mitochondrial dysfunction, drug repurposing, nanotechnology

## Abstract

Mitochondria are the primary source of energy production in cells, supporting the metabolic demand of tissue. The dysfunctional mitochondria are implicated in various diseases ranging from neurodegeneration to cancer. Therefore, regulating dysfunctional mitochondria offers a new therapeutic opportunity for diseases with mitochondrial dysfunction. Natural products are pleiotropic and readily obtainable sources of therapeutic agents, which have broad prospects in new drug discovery. Recently, many mitochondria-targeting natural products have been extensively studied and have shown promising pharmacological activity in regulating mitochondrial dysfunction. Hence, we summarize recent advances in natural products in targeting mitochondria and regulating mitochondrial dysfunction in this review. We discuss natural products in terms of their mechanisms on mitochondrial dysfunction, including modulating mitochondrial quality control system and regulating mitochondrial functions. In addition, we describe the future perspective and challenges in the development of mitochondria-targeting natural products, emphasizing the potential value of natural products in mitochondrial dysfunction.

## Introduction

Mitochondria are the primary source of energy production in mammalian cells and are responsible for producing adenosine triphosphate (ATP) through several enzymatic pathways, including the tricarboxylic acid (TCA) cycle, oxidative phosphorylation (OXPHOS), and fatty acid beta-oxidation (Kessous et al., 2020). Apart from energy metabolism, mitochondria also play important roles in other physiological processes, such as calcium homeostasis, reactive oxygen species (ROS) generation, and apoptotic cell death (Koch et al., 2017; Bock and Tait, 2020). Given the essential function of mitochondria, it is not surprising that mitochondrial dysfunction is implicated in various diseases ranging from neurodegeneration to cancer (Lin and Beal, 2006; Wallace, 2012). It has been reported that the functions of mitochondria are highly dependent on their structural integrity (Chan, 2020). As double-membrane structure organelles, mitochondria consist of the outer mitochondrial membrane (OMM), inner mitochondrial membrane (IMM), mitochondrial intermembrane space, and mitochondrial matrix (Frey and Mannella, 2000). Moreover, the structure and function of mitochondria are precisely regulated by multiple signaling pathways (Kummer and Ban, 2021). Thus, a more complete understanding of the structure, function, and regulation of mitochondria will be essential to promote the development of new therapeutic agents and may offer new combinatory therapeutic strategies.

Natural products are valuable sources for the discovery of new drugs, in which researchers can isolate active agents that might serve as leads or scaffolds for the construction of novel medicines (Li and Vederas, 2009; Rodrigues et al., 2016). Historically, numerous natural products isolated from various sources, including plants, animals and microorganisms, have been recognized as potential therapeutic agents (Khalifa et al., 2019). For example, plant-derived natural products such as flavonoids, alkaloids, terpenoids, and quinones are suitable to gain prominence as drug candidates for clinical therapy due to their chemical diversity and pleiotropic activities (Buyel, 2018; Zhang et al., 2021). In this context, botanical drugs have been used widely in China for thousands of years, and many prescription medicines obtained from plants have been approved by the Food and Drug Administration (FDA) in the United States (Newman and Cragg, 2016; Yuan et al., 2016). Recently, there has been a revitalization of interest in the discovery of natural products for targeting mitochondrial function and modulating mitochondrial dysfunction (Martucciello et al., 2020; Yang et al., 2020). Natural products serve as drug candidates for mitochondrial dysfunction through multifaceted pathways, including stimulating mitochondrial biogenesis, regulating mitochondrial fusion and fission, removing damaged mitochondria, improving mitochondrial bioenergetics, and modulating mitochondrial homeostasis. Therefore, natural products targeting mitochondria should be considered new opportunities for diseases related to mitochondrial dysfunction, such as neurodegenerative diseases and diabetes, with potentially higher success rates in clinical applications (Mu et al., 2020; Liang et al., 2021).

In this review, we summarize recent advances in the regulation of mitochondria by natural products, highlighting their multifaceted role in mitochondrial dysfunction. Our analysis has been based on the use of several databases (PubMed, ClinicalTrials, Google Scholar, and Web of Science), including the available data up to 2022 using the following keywords: natural products; natural compounds; mitochondria; mitochondrial dysfunction. In addition, we discuss the future perspective and challenges in developing natural products to target mitochondria. We hope this review will expand the therapeutic approach to mitochondrial diseases by using natural products based on modulating mitochondria.

## Natural products regulate mitochondrial quality control system

The quality and quantity of mitochondria are tightly regulated by a dynamic control system to maintain a healthy and functional mitochondrial network (Pickles et al., 2018). In mitochondrial control system, mitochondria are continuously formed and removed through mitochondrial biogenesis (the creation of new mitochondria), mitochondrial dynamics (the fusion and fission of the mitochondria), and mitophagy (the removal of damaged mitochondria) (Fu et al., 2019). Therefore, it is essential to elucidate how natural products regulate the mitochondrial control system to restore mitochondrial dysfunction.

### Triggering mitochondrial biogenesis

Mitochondrial biogenesis is a complex process that requires the coordinated regulation of nuclear and mitochondrial genomes to execute several processes, including the synthesis of inner and outer mitochondrial membranes, the synthesis of mitochondrial-encoded proteins, the synthesis and import of nuclear-encoded mitochondrial proteins, and the replication of mitochondrial DNA (mtDNA) (Jiang et al., 2019). Adenosine 5‘-monophosphate (AMP)-activated protein kinase (AMPK) and nicotinamide adenine dinucleotide (NAD^+^)-dependent deacetylase sirtuin-1 (SIRT1) directly activate phosphorylate peroxisome proliferator-activated receptor gamma coactivator 1 alpha (PGC-1α) through deacetylation and phosphorylation, respectively (Thomas and Ashcroft, 2019). Subsequently, activated PGC-1α binds to peroxisome proliferator-activated receptor- γ (PPARγ) and regulates the nuclear respiratory factors (NRF)-1/2, leading to the expression of mitochondrial transcription factor A (TFAM) and other nuclear-encoded mitochondrial proteins (Popov, 2020). Therefore, identifying drug candidates that modulate the AMPK-SIRT1-PGC-1α pathway may be beneficial for mitochondrial dysfunction through stimulating mitochondrial biogenesis.

Many polyphenolic compounds from natural plants have been reported to activate AMPK and upregulate mitochondrial biogenesis (Biasutto et al., 2011). Green tea (*Camellia sinensis* L. (Kuntze)) has been demonstrated to possess many health benefits, which are mainly attributed to the polyphenolic components with antioxidant properties (Prasanth et al., 2019). Epigallocatechin-3-gallate (EGCG), a flavone-3-ol polyphenol, is the major bioactive component in green tea (Gan et al., 2018). Xiong et al. (2018) have demonstrated that EGCG increases the levels of AMPK and SIRT1 along with triggering mitochondrial biogenesis. In cells from subjects with Down syndrome, EGCG is a promoting regulator of mitochondrial biogenesis by increasing SIRT1-dependent PGC-1α deacetylation (Valenti et al., 2013). Thus, EGCG may have therapeutic potential for Down syndrome by increasing mitochondrial biogenesis with a mechanism involving the SIRT1-dependent PGC-1α-NRF-1/2-TFAM pathway. Similarly, the natural polyphenol resveratrol triggers mitochondrial biogenesis and restores oxidative phosphorylation efficiency in the Down syndrome model (Valenti et al., 2016). Furthermore, resveratrol has been shown to stimulate mitochondrial biogenesis and induces beneficial effects on mitochondrial function in experimental models of neurodegenerative disorders, such as Parkinson’s disease (PD) and Alzheimer’s disease (AD) (Ferretta et al., 2014; Porquet et al., 2014). Resveratrol, a naturally occurring polyphenolic compound, is mainly present in grapes (*Vitis vinifera* L. (Vitaceae)) as well as in red wine (Rauf et al., 2018). According to previous studies, resveratrol and its analogs are extensively utilized as antioxidants, anti-inflammatory, and anticancer agents (Ren B. et al., 2021; Zhou et al., 2021). Moreover, resveratrol has been reported that play multifaceted roles in modulating mitochondrial function in various *in vitro* and *in vivo* models (Jardim et al., 2018). In terms of mechanism, resveratrol triggers mitochondrial biogenesis through the activation of the AMPK/SIRT1/PGC-1α axis (Lagouge et al., 2006; de Oliveira et al., 2016). Notably, SIRT1-knockout mice show no increase in AMPK activation and mitochondrial biogenesis after treating a moderate dose of resveratrol, whereas SIRT1 overexpressing obviously increases these effects, suggesting that SIRT1 is required for AMPK activation and the beneficial effects of resveratrol on mitochondrial function (Price et al., 2012). Accordingly, the use of natural products polyphenol as alternative drugs for triggering mitochondrial biogenesis is a potential therapeutic strategy in preventing or managing neurodegenerative disease.

Apart from polyphenolic compounds, alkaloids were noted for their pharmacological activities in triggering mitochondrial biogenesis. For example, natural alkaloid bouchardatine increased SIRT1 activity to activate the liver kinase B1 (LKB1)-mediated AMPK, thereby promoting mitochondrial biogenesis in adipose tissues and finally ameliorating obesity-related metabolic disorders (Rao et al., 2017). Berberine, an isoquinoline alkaloid from Barberry (*Berberis vulgaris* L.), protects against high-fat diet-induced dysfunction in muscle cells *via* stimulating SIRT1-dependent mitochondrial biogenesis (Gomes et al., 2012; Imenshahidi and Hosseinzadeh, 2016). Songorine, a typical diterpene alkaloid from the lateral root of *Aconitum carmichaelii* Debx. (Ranunculaceae), promotes cardiac mitochondrial biogenesis during sepsis through activating the NRF2/ARE and NRF1 signaling cascades (Li Y. et al., 2021).

**FIGURE 1:**
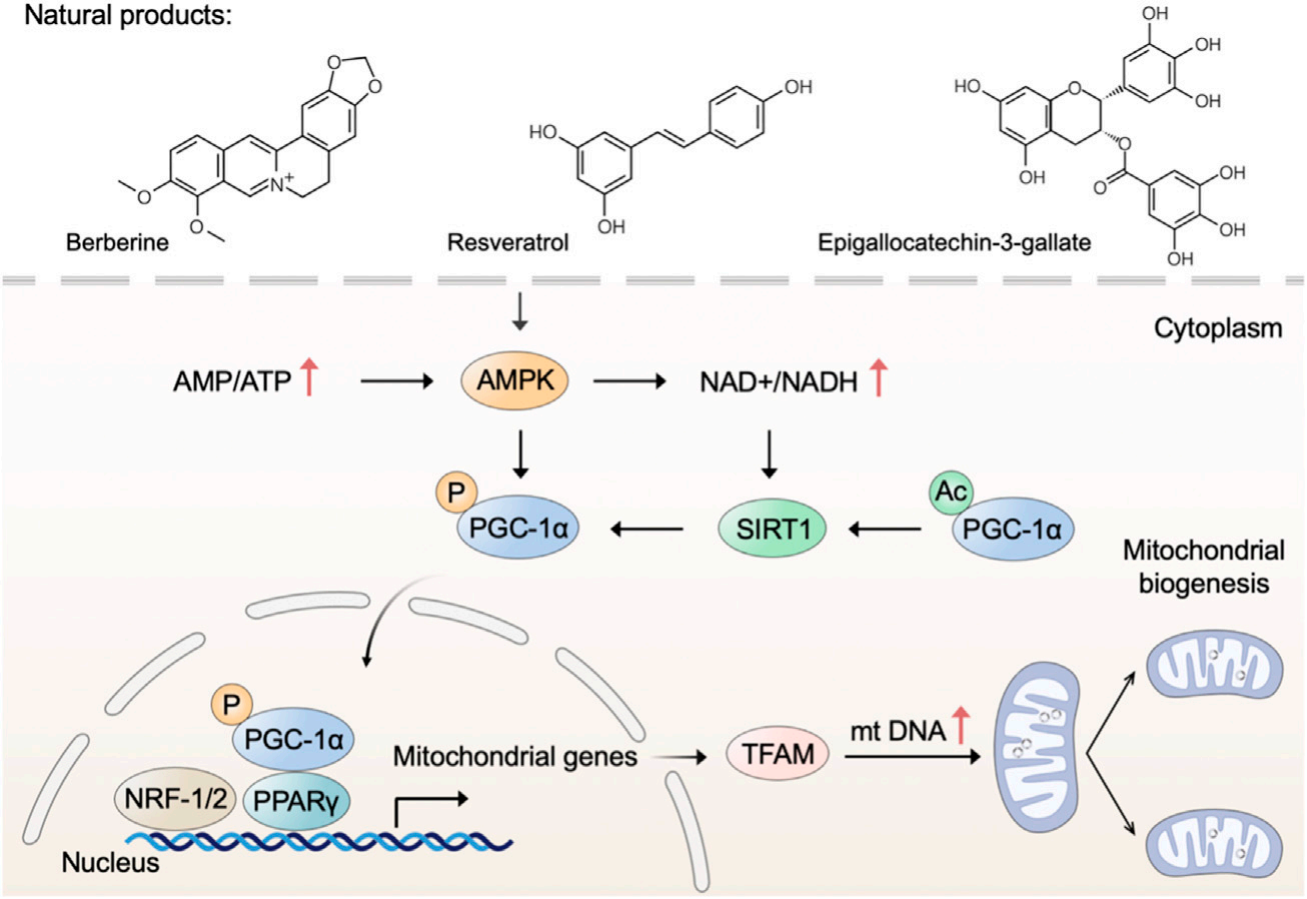
Natural products trigger mitochondrial biogenesis. Natural products such as berberine, resveratrol, and epigallocatechin-3-gallate trigger mitochondrial biogenesis through regulating the AMPK-SIRT1-PGC-1α pathway.

In addition, a growing number of other natural products, such as astaxanthin (Nishida et al., 2020), ginsenoside (Huang et al., 2021), corylin (Chen C.-C. et al., 2021), and betaine (Ma et al., 2019), have been reported that possess the capability to trigger mitochondrial biogenesis. Taken together, natural products with anticipated safety are expected to be developed into long-term dietary supplements to prevent or treat mitochondrial dysfunction through mechanisms involving mitochondrial biogenesis (Figure 1).

### Promoting mitochondrial fusion and fission

Mitochondria are highly dynamic organelles that continuously undergo fission and fusion, a process called mitochondrial dynamics (Tilokani et al., 2018). As a critical process in maintaining mitochondrial morphology and homeostasis, mitochondrial dynamics are important in the health of mitochondria and cells (Chan, 2020). Specifically, mitochondrial fusion accelerates the material exchange in mitochondria to compensate for mitochondrial function, and mitochondrial fission removes damaged mitochondria through mitophagy (Adebayo et al., 2021). In mammalian cells, the balance in mitochondrial fusion-fission is regulated by various regulators (van der Bliek et al., 2013). Mitochondrial fusion in outer mitochondrial membrane is mediated by mitofusins 1 and 2 (MFN1 and MFN2), while fusion in inner mitochondrial membrane is mediated by optic atrophy protein 1 (OPA1) (Gao and Hu, 2021). And mitochondrial fission is mediated by dynamin-related protein 1 (DRP1), mitochondrial fission protein 1 (FIS1) and mitochondrial fission factor (MFF) (Otera et al., 2013). Research revealed that mitochondrial dysfunction caused by defects in mitochondrial dynamics is implicated in many diseases, such as neurodegeneration and cardiac disease (Forte et al., 2021; Coelho et al., 2022). Thus, developing natural products with pharmacological effects on mitochondrial fusion and fission represents an attractive strategy to intervene in mitochondrial dysfunction.

There is a bulk of available evidence that natural polyphenol curcumin and its derivatives can regulate mitochondrial dynamics and, as a consequence, remedy mitochondrial dysfunction. Curcumin, an active component found in the rhizome of *Curcuma longa* L. (Zingiberaceae), has shown promising protective effects in several disease models (Kocaadam and Şanlier, 2017). Indeed, curcumin prevented the increase of FIS1 and the decrease of OPA1 to restrain cisplatin-induced renal alterations (Ortega-Domínguez et al., 2017). Contradictorily, Eckert *et al.* have reported that curcumin enhances mitochondrial fission by regulating the expression of FIS1 and DRP1 and restores mitochondrial fusion in brains of SAMP8 mice (Eckert et al., 2013). As a natural derivative of curcumin, tetrahydrocurcumin, was found to regulate mitochondrial dynamics through upregulated fission marker (DRP1) and fusion marker (MFN2), thereby ameliorating homocysteine-mediated mitochondrial remodeling in brain endothelial cells (Vacek et al., 2018).

Flavonoids are the widest group of natural polyphenolic compounds, which can be divided into different subgroups: flavones, flavonols, flavanones, isoflavone, anthocyanins and chalcones (Zhang et al., 2021). As the great representative of flavonols, quercetin has prophylactic potential for the amelioration of hypobaric hypoxia-induced memory impairment, which is partly attributed to its ability to regulate mitochondrial dynamics (Liu et al., 2015). In terms of mechanism, quercetin inhibits fission by decreasing the expression of DRP1 and FIS1, and simultaneously enhances fusion by increasing the expression of MFN1 and MFN2 (Liu et al., 2015). Differently, hyperoside, a natural flavonol glycoside in the flowers of *Abelmoschus manihot* L. (Malvaceae), was found to inhibit ischemia/reperfusion-induced mitochondrial fission by suppressing OMA1 mediated proteolysis of OPA1 (Wu et al., 2019). The flavone xanthohumol is a natural product derived from *Humulus lupulus* L. (Cannabaceae), which has been reported to upregulate the expression of MFN2 to promote mitochondrial fusion and alleviate excitotoxicity in the rat brain (Wang et al., 2020). Moreover, the flavanone liquiritigenin extracted from the radix of *Glycyrrhiza uralensis* Fisch. (Fabaceae) was found to regulate mitochondrial dynamics and prevent beta-amyloid (Aβ) -induced neurotoxicity (Jo et al., 2016). In human SK-N-MC cells, liquiritigenin induced an elongated mitochondrial morphology and rescued mitochondrial fragmentation caused by the knockout of fusion markers, including MFN1, MFN2, and OPA1.

In addition, gastrodin, the main bioactive component of *Gastrodia elata* Bl. (Orchidaceae), was found to reverse the dysregulation of mitochondrial fusion and fission mediators for maintaining the structure and functions of mitochondria (Huang et al., 2019; Cheng et al., 2020). And an increasing number of other natural products, such as cryptotanshinone (Yen et al., 2019), grape seed proanthocyanidins (Yang et al., 2017), panaxadiol ginsenoside (Dong et al., 2016), rosmarinic acid (Gupta et al., 2022), ilexgenin A (Zhu et al., 2019), lycium barbarum polysaccharide (Li et al., 2022), and extracts from *Rhodiola crenulata* (Hook. f. et Thoms.) H. Ohba (Crassulaceae) (Dun et al., 2017), have been reported to regulate mitochondrial dynamics through modulating mitochondrial fusion and fission mediators.

**FIGURE 2:**
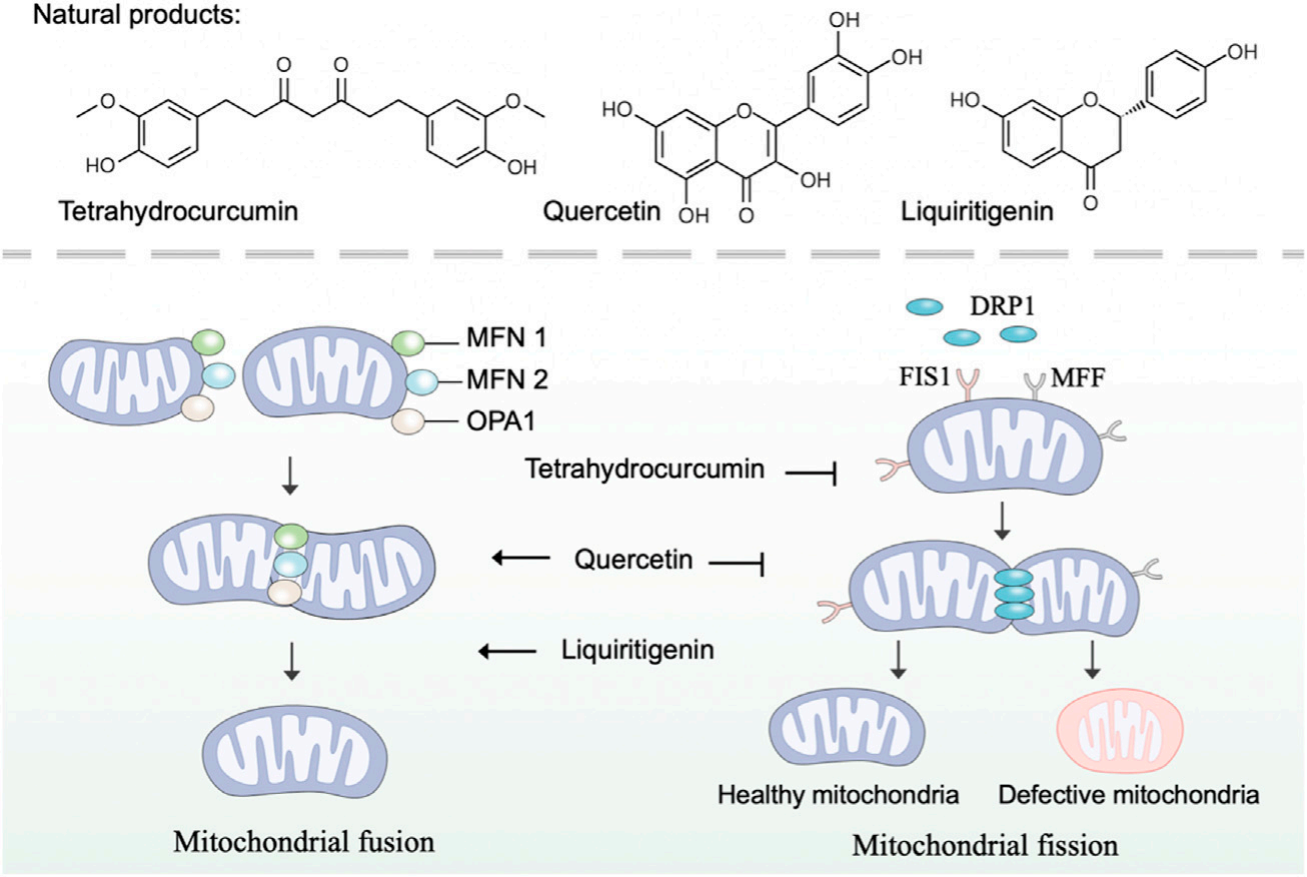
Natural products regulate mitochondrial fusion and fission. Mitochondrial fusion is mediated by MFN1, MFN2, and OPA1, while mitochondrial fission is mediated by DRP1, FIS1, and MFF. Natural products such as tetrahydrocurcumin, quercetin, and liquiritigenin regulate mitochondrial dynamics by modulating mitochondrial fusion and fission mediators.

Taken together, many natural products have been reported to regulate mitochondrial fusion and fission, thereby possessing promising prophylactic effects in several disease models (Figure 2). Nevertheless, few natural products individually target mitochondrial fusion or fission markers, which poses a selective challenge for using natural products to specifically modulate mitochondrial dysfunction for the treatment of related diseases. Noticeably, the regulatory roles of some natural products, such as quercetin, in mitochondrial dynamics were found to be inconsistent in different disease models, suggesting more preclinical trials are needed in the future to explore the regulation of natural products on mitochondrial dysfunction.

### Inducing mitophagy

Mitophagy is an evolutionarily conserved cellular process to control mitochondrial quality through degrading dysfunctional or damaged mitochondria (Pickles et al., 2018). As a selective form of autophagy, mitophagy selectively targets unhealthy mitochondria or aberrant mitochondrial proteins to autophagosomes for degradation (Ashrafi and Schwarz, 2013). In general, mitophagy could be categorized into two main categories: PINK1/Parkin (PTEN Induced Kinase 1/Parkin RBR E3 Ubiquitin Protein Ligase)-mediated mitophagy and receptor-mediated mitophagy (Onishi et al., 2021). Under normal conditions, PINK1 is imported into mitochondria to be cleaved by proteases and subsequently degraded by the ubiquitin-proteasome system (Nguyen et al., 2016). However, in damaged mitochondria that lose mitochondrial membrane potential, PINK1 ceases to be imported and instead accumulates on the mitochondrial outer membrane (MOM) (Pickrell and Youle, 2015). The accumulated PINK1 phosphorylates E3-ubiquitin ligase Parkin and activates Parkin-mediated ubiquitination to drive mitophagy (Eiyama and Okamoto, 2015; Agarwal and Muqit, 2022). Receptor-mediated mitophagy relies on multiple receptors (BNIP3, NIX, FUNDC1, BCL2L13, FKBP8) that anchor in the MOM *via* their C-terminal transmembrane domains and interacts with microtubule-associated protein light chain 3 (LC3) *via* their LIR (LC3-interacting region) motif (Liu et al., 2014). Once mitophagy is activated, these receptors directly bind LC3 in a ubiquitin-independent manner to bring together autophagosomal membranes and mitochondria for the degradation of target mitochondria (Yamaguchi et al., 2016). In brief, inducing mitophagy that results in the elimination of damaged mitochondria by utilizing natural products is a promising approach to combat mitochondrial dysfunction.

Over the past few years, several plant-derived compounds have been found to induce mitophagy. The natural flavone acacetin has been demonstrated to enhance mitophagy and preserve mitochondrial function for alleviating cardiac senescence in a concentration-dependent manner. In aging mice with the treatment of oral acacetin, the increased expression of cellular senescence marker proteins (p21 and p53) and the reduced expression of mitophagy signaling proteins (PINK1 and Parkin) were reversed (Hong et al., 2021). Similarly, as an important organosulfur compound derived from garlic, alliin was found to promote mitophagy through the PINK 1/Parkin pathway. The PINK 1/Parkin-mediated mitophagy induced by alliin can reduce intracellular ROS, thus relieving lipopolysaccharide-induced pyroptosis (Liu M. et al., 2022). Polydatin, a polyphenol extracted from the rhizome of *Polygonum cuspidatum* Sieb. et Zucc. (Polygonaceae), has anti-oxidative and anti-inflammatory effects (Zhao et al., 2018). Li *et al.* have found that polydatin can facilitate Parkin translocation to mitochondria and activate Parkin-dependent mitophagy in acute respiratory distress syndrome (Li et al., 2019). Consistently, in Parkin-knockout mice and Parkin siRNA transfected cells, polydatin-induced mitophagy was inhibited. Furthermore, polydatin protected against sepsis-induced acute kidney injury, and the underlying mechanisms include the activated Parkin-dependent mitophagy and the suppressed NOD-like receptor thermal protein domain associated protein 3 (NLRP3) inflammasome (Gao et al., 2020).

Several plant extracts have been reported to stimulate mitophagy and may confer a variety of health benefits. Upon onset of stress conditions, pomegranate extract activates transcription factor EB to upregulate the expression of autophagy and lysosomal genes for mitophagy (Tan et al., 2019). Furthermore, pomegranate extract engages PINK1 and Parkin to the mitochondria, and simultaneously augments mitophagosome formation to potentiate mitophagy (Tan et al., 2019). The protective effects of pomegranate extract-induced mitophagy include the elimination of superfluous mitochondrial ROS and alleviation of mitochondrial dysfunction, suggesting that pomegranate extract supplementation is expected to prevent mitochondria-related diseases. Moreover, artemisia leaf extract and grape skin extract were found to exert neuroprotective effects by inducing mitophagy in both *in vitro* and *in vivo* models (Wu et al., 2018; Wu et al., 2022).

In addition to plant-derived natural products, other natural products such as spermidine and gramicidin A are also attractive in inducing mitophagy. Spermidine is a natural metabolite that has been reported to be involved in maintaining cellular homeostasis and preserving mitochondrial function (Madeo et al., 2018). Eisenberg *et al.* have investigated the cardioprotective effect of oral spermidine in mice, and they have found that spermidine feeding enhanced cardiac autophagy, mitophagy and mitochondrial respiration, and improved the mechano-elastical properties of cardiomyocytes. Gramicidin A is a linear 15-mer peptidic natural product that has been followed with interest as a cytostatic agent (Xue et al., 2022). Xue *et al.* have revealed that gramicidin A accumulates in mitochondria, reduces ATP levels, induces mitophagy, and ultimately leads to potent inhibition of cell growth.

**FIGURE 3:**
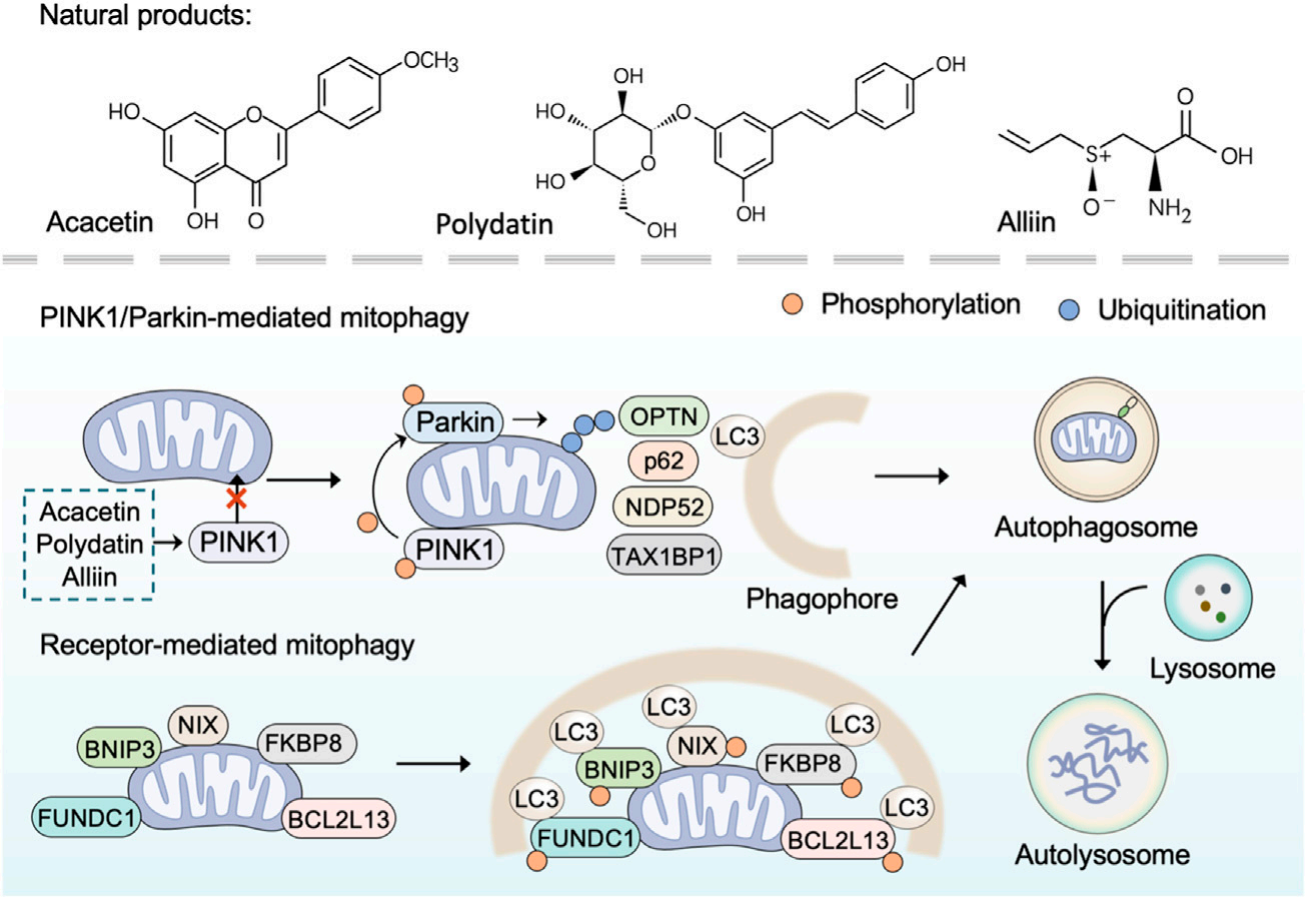
Natural products induce mitophagy. PINK1/Parkin-dependent mitophagy is mediated by the accumulated PINK1 through phosphorylating E3-ubiquitin ligase Parkin and activating Parkin-mediated ubiquitination to recruit adaptor proteins (such as p62, OPTN, TAX1BP1, NDP52). The adaptor proteins then bind phosphorylated poly-ubiquitinated chains on mitochondria and LC3, and thus lead to the engulfment of target mitochondria within autophagosome for final degradation Receptor-mediated mitophagy is mediated by receptor proteins (BNIP3, NIX, FUNDC1, BCL2L13, FKBP8) through binding LC3 directly in a ubiquitin-independent manner to bring together autophagosomal membranes and mitochondria for the degradation of target mitochondria. Natural products such as polydatin, acacetin, and alliin induce mitophagy through the PINK 1/Parkin pathway.

Overall, many natural products, especially plant-derived compounds, have been reported to regulate mitophagy, suggesting their therapeutic potential for overcoming mitochondrial disorders (Figure 3). Most of these natural products induce mitophagy through the PINK 1/Parkin pathway. However, there are still a few natural products that have been reported to inhibit mitophagy. For instance, salvianolic acid B protects against endothelial dysfunction by inhibiting Rho-associated protein kinase 1 (ROCK1)-mediated mitophagy and apoptosis (Ko et al., 2020).

## Natural products regulate mitochondrial functions

Most natural products modulate mitochondrial dysfunction by directly maintaining and promoting mitochondrial functions. Although mitochondria remain best known for their role in bioenergetics, they are increasingly being recognized for their role in signaling events (Chandel, 2015). Herein, we respectively describe natural products targeting mitochondria in bioenergetics, calcium buffering, and cell death.

### Regulating mitochondria in cellular bioenergetics

Cellular bioenergetics refers to the biochemical and molecular processes involved in energy metabolism, which are largely affected by mitochondria (Acuña-Castroviejo et al., 2001). As bioenergetic and biosynthetic organelles, the most prominent role of mitochondria is to generate energy by OXPHOS and support anabolism by the TCA cycle (also referred to as the Krebs cycle or the citric acid cycle) (Spinelli and Haigis, 2018). In mammalian cells, the glycolysis-derived pyruvate is predominantly imported into the mitochondria after decarboxylation by the pyruvate dehydrogenase (PHD) complex to form acetyl coenzyme A (acetyl-CoA) (McCommis and Finck, 2015). Acetyl-CoA fuels the TCA cycle to reduce NAD^+^ and adenine dinucleotide (FAD) to NADH and FADH_2_, respectively, which are subsequently used as substrates of the electron transport chain to generate ATP *via* OXPHOS (Fernie et al., 2004). The mitochondrial OXPHOS system comprises two mobile electron carriers (ubiquinone and cytochrome c) and five enzymatic complexes, including complex I (NADH dehydrogenase), complex II (succinate dehydrogenase), complex III (cytochrome bc1 complex), complex IV (cytochrome c oxidase), and complex V (ATP synthase) (Letts and Sazanov, 2017). Complexes I to IV constitute the mitochondrial respiratory chain (MRC), which generates a proton gradient across the inner mitochondrial membrane, and complex V subsequently couples proton reflux to generate ATP from adenosine diphosphate (ADP) and phosphate (Vercellino and Sazanov, 2022). The ATP generated from mitochondria is exported to the cytosol, where it is hydrolyzed to ADP to fuel various vital cellular processes (Boyman et al., 2020). Previous studies have suggested that minimal perturbations in cellular bioenergetics are linked to diseases, highlighting the potential to target mitochondria in bioenergetics and metabolic regulation (Protasoni and Zeviani, 2021).

Bioactive ingredients from magnolia extracts, such as magnolol, honokiol, and 4-O-methylhonokiol, have received extensive attention for their chemopreventive effects (Lee et al., 2011). Zhang et al. (2020) have found that magnolia extracts inhibit mitochondrial respiration at complex I of the electron transport chain, oxidize peroxiredoxins, activate AMPK, and inhibit signal transducer and activator of transcription 3 (STAT3) phosphorylation. Meanwhile, magnolia extracts did not cause detectable side effects in animal models (Zhang et al., 2020). These results provide novel information on the mechanism of magnolia extract in oral cancer cells and confirm the usefulness of magnolia extracts as a safe chemopreventive agent.

Due to its multi-targeting modes of action, pharmacological modulation of some natural products affects various aspects of mitochondrial function. As mentioned above, resveratrol is a multifunctional natural compound that triggers mitochondrial biogenesis and induces cell death. Furthermore, resveratrol supplementation has been shown to induce beneficial metabolic effects (Schulz et al., 2014). Indeed, Timmers et al. (2011) have demonstrated that resveratrol activated AMPK, increased SIRT1 and PGC-1α protein levels without changes in mitochondrial content, and improved mitochondrial respiration on a fatty acid-derived substrate. Regarding the extract from bergamot (*Citrus bergamia* L. (Rutaceae)), it eradicates cancer stem cells by targeting mevalonate, Rho-GDI-signaling and mitochondrial metabolism (Fiorillo et al., 2018). In detail, the extract from bergamot inhibits the OXPHOS system and fatty acid oxidation (FAO) to reduce mitochondrial metabolism.

Intracellular energy produced through mitochondrial respiration can be used to promote neuron viability and enhance synaptic plasticity (Kann and Kovács, 2007; Rossoll and Bassell, 2019). Thus, natural products that modulate mitochondrial respiration appear to be promising drugs for preventing neurotoxicity or overcoming neurodegeneration. For instance, schisandra extract and ascorbic acid synergistically increase basal oxygen consumption rate in mouse hippocampal cells and enhance cognition in mice through modulating mitochondrial respiration (Jang et al., 2020). Baicalein prevented rotenone-induced ATP deficiency in both isolated rat brain mitochondria and PC12 cells by promoting mitochondrial respiration (Li et al., 2012). Gastrodin possesses the ability to cross the blood-brain barrier and has been proven to alleviate cognitive impairment in experimental animals (Wan et al., 2020). Moreover, gastrodin exerts neuroprotective effects against H_2_O_2_-induced oxidative stress in human SH-SY5Y neuroblastoma cells, where it increases mitochondrial respiration (de Oliveira et al., 2018).

**FIGURE 4:**
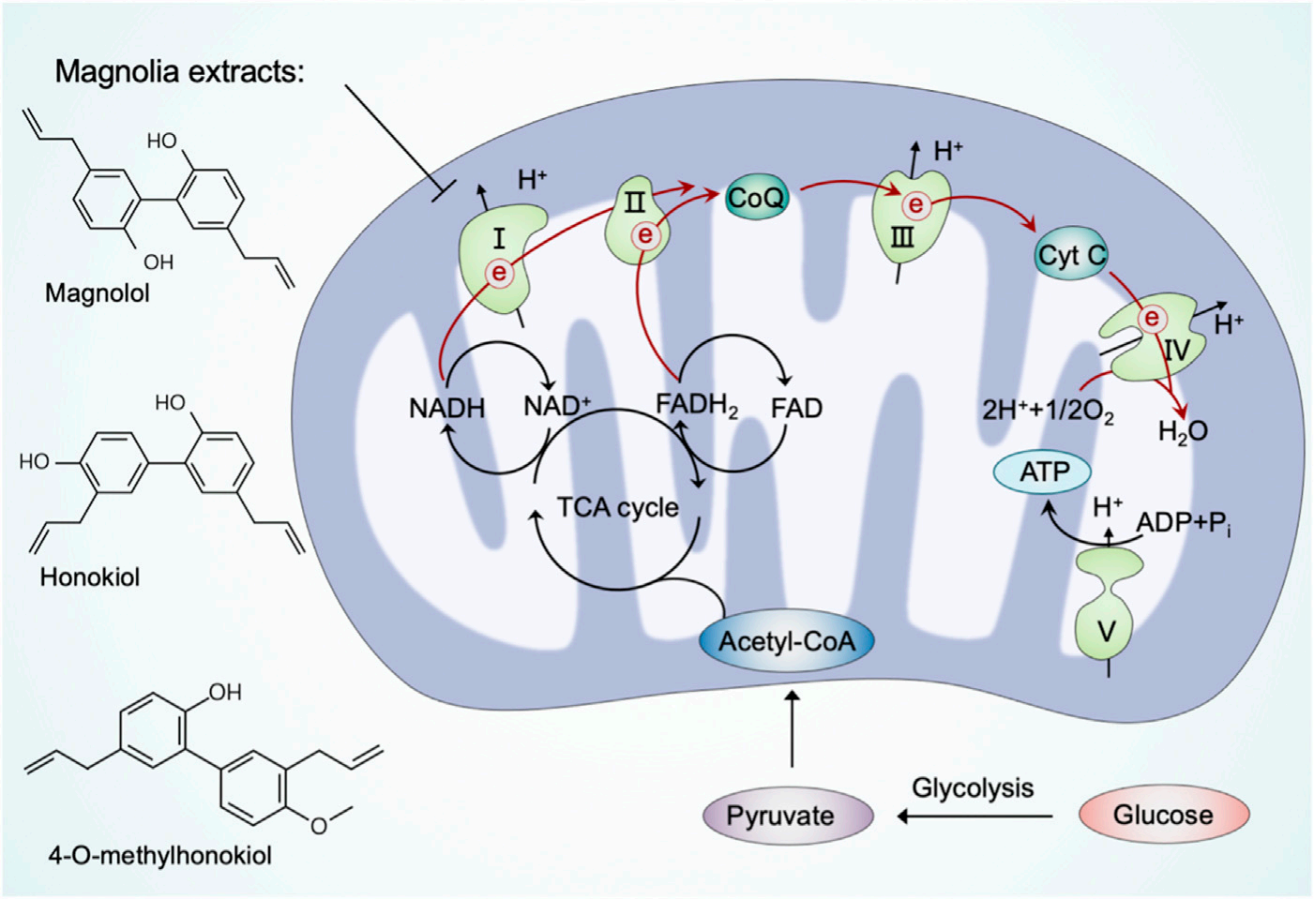
Natural products regulate mitochondria in cellular bioenergetics. Pyruvate, the end-product of glycolysis, is predominantly imported into mitochondria to form acetyl coenzyme A (acetyl-CoA) by the pyruvate dehydrogenase (PDH) complex. Acetyl-CoA fuels the TCA cycle to reduce NAD^+^ and FAD to NADH and FADH_2_, respectively, which subsequently power up ATP production through the electron transport chain. Natural products, such as magnolia extracts, inhibit mitochondrial respiration at complex I to reduce cellular bioenergetics.

In summary, an increasing number of natural products have been reported in recent years to modulate mitochondrial respiration, thereby contributing to neuroprotection (Figure 4). Notably, some of these natural products share structural characteristics with the aforementioned natural compounds that regulate mitochondrial quality control systems.

### Regulating mitochondria in calcium buffering

Calcium is utilized by cells as a second messenger, which is crucial to a myriad of cellular processes ranging from metabolic regulation to vesicle release (Snoeck, 2020). Mitochondrial Ca^2+^ uptake and release were shown to alter the spatial and temporal profile of the intracellular Ca^2+^ signal by buffering cytosolic Ca^2+^ levels and regulating mitochondrial effectors (Rizzuto et al., 2012). Once Ca^2+^ levels rise abnormally, the voltage-dependent anion channel (VDAC) in the outer mitochondrial membrane (OMM) and the mitochondrial calcium uniporter (MCU) channels in the inner mitochondrial membrane (IMM) mediate the transfer of Ca^2+^ from the endoplasmic reticulum and cytosol to mitochondria (Drago et al., 2011). Export of Ca^2+^ from mitochondria occurs mainly through Na^+^-dependent (Na^+^/Ca^2+^/Li^+^ exchanger, NCLX) and Na^+^-independent (H^+^/Ca^2+^ exchanger, HCX) mechanisms (Modesti et al., 2021). Moreover, mitochondrial membrane potential (MMP, ΔΨ) generated by the electron transport chain represents the driving force for mitochondrial Ca^2+^ uptake (Giorgi et al., 2018). Accumulating evidence has demonstrated that mitochondrial dysfunction in calcium buffering led to various pathological conditions; thus, we discuss the possible beneficial effects of natural products targeting mitochondrial Ca^2+^ uptake and release as a strategy for modulating mitochondrial dysfunction.

Several natural products target mitochondrial Ca^2+^ uptake and release in neurodegenerative disease models. Indeed, natural products such as kaurane-type diterpenes, baicalein, and tetrahydrohyperforin were shown neuroprotective effect through mitochondria-mediated calcium homeostasis (Zolezzi et al., 2013; González-Burgos et al., 2016; Wang et al., 2017). Moreover, extracts of *sedum takesimense* (Crassulaceae) were reported to stabilize MMP and induce the closure of mitochondrial permeability transition pores to attenuate mitochondrial Ca^2+^ release, thereby protecting PC12 cells against corticosterone-induced neurotoxicity (Yun and Jeong, 2020). Similarly, catechins extracted from tea were found to rescue the decrease of MMP and the dysfunction of mitochondria for calcium buffering in PC12 cells exposed to lead.

Many polyphenolic compounds derived from plants possess modulatory roles in targeting mitochondria-mediated calcium buffering. For instance, mangiferin and morin, two natural polyphenols with antioxidant properties, were reported to scavenge ROS and promote calcium homeostasis modulated by mitochondria (Ibarretxe et al., 2006). The polyphenolic extracts from *Citrus bergamia* L. (Rutaceae) were found to reverse mitochondrial dysfunction and prevent the consequent increase in cytosolic calcium, thereby reducing sarcoplasmic reticulum stress in diabetic cardiomyopathy (Maiuolo et al., 2021). Kaempferol, a dietary polyphenol with several pharmacological properties, is being applied in chemotherapy against cancer (Imran et al., 2019). Montero et al. (2004) have found that the plant product kaempferol directly activates MCU without phosphorylation to promote mitochondrial Ca^2+^ uptake (). Subsequently, Bermont et al. (2020) have demonstrated that kaempferol supports mitochondrial Ca^2+^ uptake to promote metabolism/secretion coupling in pancreatic β-cells. Moreover, the common polyphenols quercetin, resveratrol, and rutin were shown the inhibition activity on indomethacin-induced Ca^2+^ efflux from the endoplasmic reticulum (ER) and Ca^2+^ extrusion into mitochondria; among them, quercetin has the strongest effect on preventing Ca^2+^ mobilization and subsequent cytotoxicity (Carrasco-Pozo et al., 2012).

It is worth noting that the effect of resveratrol on calcium flux is complex (McCalley et al., 2014). Although resveratrol represents positive effects on mitochondria mediated calcium buffering as described above, there are several reports about the detrimental effects on resveratrol eliciting mitochondrial Ca^2+^-overload in different experimental models (de Oliveira et al., 2016). In senescent cells, resveratrol was demonstrated to trigger cell death through mitochondrial Ca^2+^-overload exclusively (Madreiter-Sokolowski et al., 2019). In HeLa cells, resveratrol was reported to promote Ca^2+^-overload, caspase(−3, 8, 9) expression and DNA damage, leading to apoptotic cell death (Devi et al., 2021). Based on this, Devi et al. (2021) have proposed that integrating siNCLX mediated gene silencing with resveratrol is a promising synergistic therapeutic approach.

Apart from resveratrol, several natural products were shown in disrupting calcium homeostasis *via* mitochondrial dysfunction. β-sitosterol, a phytosterol, was reported to promote cell death in ovarian cancer by triggering pro-apoptosis signals and the loss of mitochondrial membrane potential and enhancing the calcium influx through the ER-mitochondria axis (Bae et al., 2021b). Polydatin was found to trigger the disturbance of mitochondrial functions in colon cancer cells by oxidative stress and the loss of mitochondrial membrane potential. Furthermore, research has shown that polydatin can induce apoptosis *via* mitochondrial dysfunction-mediated calcium influx, and the combination of polydatin with 5-Fluorouracil (5-FU) can counteract the resistance of 5-FU-resistant cells (Bae et al., 2021a). Accordingly, the regulation of mitochondria in calcium homeostasis has been expected as a new anticancer strategy, and several natural products that target mitochondria are applied in promoting Ca^2+^-overload and the inhibition of tumors.

**FIGURE 5:**
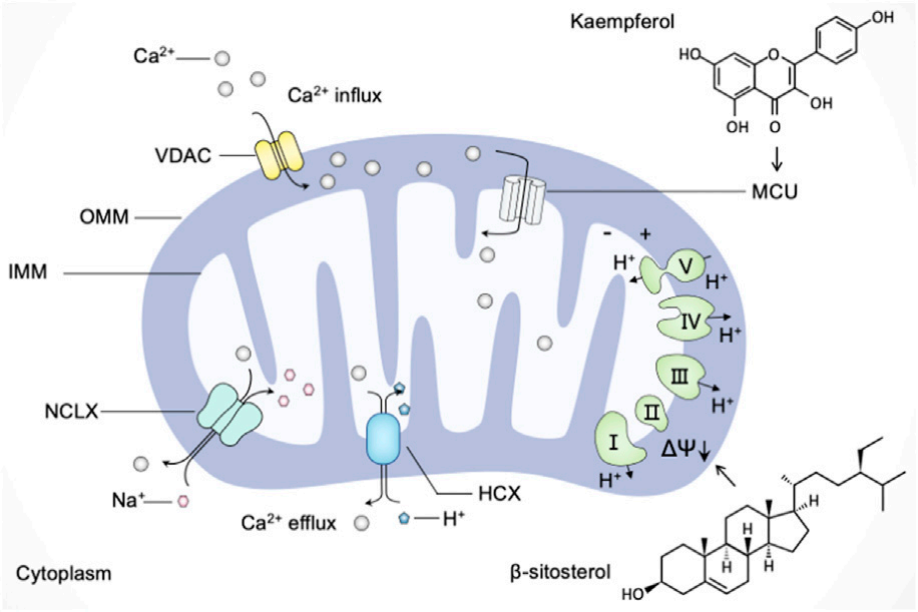
Natural products regulate mitochondria in calcium buffering. Ca^2+^ enters mitochondria through the voltage-dependent anion channel (VDAC) in the outer mitochondrial membrane (OMM) and the mitochondrial calcium uniporter (MCU) channels in the inner mitochondrial membrane (IMM). Ca^2+^ exports from mitochondria towards the cytoplasm mainly through the Na^+^/Ca^2+^/Li^+^ exchanger (NCLX) and H^+^/Ca^2+^ exchanger (HCX). The driving force for mitochondrial Ca^2+^ mobilization is mediated by mitochondrial membrane potential (ΔΨ). Natural products such as kaempferol and β-sitosterol regulate mitochondria in calcium buffering in different manners. Kaempferol directly activates MCU to promote mitochondrial Ca^2+^ uptake, and β-sitosterol triggers the loss of mitochondrial membrane potential and enhance Ca^2+^ calcium influx.

In summary, these findings advance our understanding of natural products' complex impact on mitochondria in calcium regulation and contribute to the concept of natural products as pleiotropic agents in nutritional intervention strategy (Figure 5). As mentioned above, mitochondrial Ca^2+^-overload is detrimental to the neuron and has been implicated in neurodegeneration, while excessive mitochondrial Ca^2+^ can induce apoptosis in cancer cells and make cancer cells more susceptible to chemotherapeutic drugs. Therefore, it is necessary to evaluate the modulatory roles of different natural products in various disease models to propose a reasonable regimen in different clinical settings.

### Regulating mitochondria in cell death

Although essential for cell sustenance, paradoxically, mitochondria have a central role in initiating cell death (Jeong and Seol, 2008; Tait and Green, 2013). Mitochondrial apoptosis is mediated by mitochondrial outer membrane permeabilization (MOMP), a defining event representing irreversible cell death (Bender and Martinou, 2013). Following MOMP, multiple pro-apoptotic factors, notably cytochrome C, are released from mitochondria into the cytosol and bind to the adaptor molecule apoptotic peptidase activating factor 1 (APAF1) to form the apoptosome, thereby triggering the activation of the caspase-dependent mitochondrial apoptotic pathway (Kroemer et al., 2007). Simultaneously, MOMP releases other proteins, including SMAC and OMI, that block the caspase inhibitor XIAP (X-linked inhibitor-of-apoptosis protein), thereby promoting apoptosis (Tait and Green, 2010).

In addition to apoptotic cell death, mitochondria can induce cellular oxidative stress and even ferroptosis due to their ability to generate ROS (Zorov et al., 2014; Wang et al., 2021). In detail, the electron leakage from the electron transport chain complexes I and III results in the intracellular production of superoxide (O_2_^−^) that is subsequently converted to hydrogen peroxide (H_2_O_2_) through superoxide dismutase (SOD)-mediated disproportionation reaction (Turrens, 2003). H_2_O_2_ can be further catalyzed to H_2_O and O_2_ *via* antioxidant enzymes such as catalase (CAT), peroxiredoxins (PRX), and glutathione peroxidase (GPX) (Bhatti et al., 2017). On the other hand, H_2_O_2_ can be converted to hydroxyl radical (•OH) *via* Fenton reaction in the presence of metal ions such as Fe^2+^ and Cu^2+^ (Li X. et al., 2021). Excessive ROS (including O_2_^−^, H_2_O_2_, and •OH) can trigger DNA damage, protein fragmentation, and lipid peroxidation to induce cell death (Sena and Chandel, 2012). Current literature studies revealed that mitochondria induce ferroptotic cell death through multifaceted mechanisms (Wu et al., 2021). Ferroptosis is an iron-dependent, non-apoptotic pathway of regulated cell death that is driven by the toxic build-up of lipid peroxides and is controlled by integrated oxidation and antioxidant systems (Chen X. et al., 2021; Lei et al., 2022). The cystine/glutamate antiporter (system Xc-), glutathione peroxidase 4 (GPX4), and mitochondria have been identified as key nodes in ferroptosis pathway (Tang et al., 2021; Wu et al., 2021). In-depth study of mitochondria-mediated cell death sheds new light on eliminating cancer cells (Bock and Tait, 2020). Thus, screening and developing natural products that target mitochondria-mediated cell death represent attractive strategies for treating various cancers (Yang et al., 2020).

In acute myeloid leukemia cells, plant-derived products, such as catechins, camptothecin, and khat can induce apoptosis, which is associated with mitochondrial damage and caspase activation (Bredholt et al., 2009; Zhang et al., 2014). Notably, catechins inhibit primary acute myeloid leukemia cells without affecting normal hematopoietic progenitor cells, suggesting that catechins may be promising chemotherapeutic agents for treating acute myeloid leukemia or other hematological malignancies (Zhang et al., 2014). In human cutaneous squamous cell (SCC) carcinoma cells, vanilloids at micromolar concentration levels induce apoptosis, which appears to involve the permeability of the inner mitochondrial membrane and the inhibition of mitochondrial respiration (Hail and Lotan, 2002). In multiple myeloma, baicalein inhibits proliferation and induces mitochondria-mediated apoptosis of human myeloma cells *via* reducing mitochondrial membrane potential and activating caspase 9 and caspase 3 (Ma et al., 2005). In hepatoblastoma, icariside II induces mitochondrial and lysosomal membrane permeabilization, resulting in leakage of the hydrolases from lysosomes and pro-apoptosis members from mitochondria (Geng et al., 2015). And in colorectal cancer cells, American ginseng root extracts induce mitochondrial damage and ROS surge to mediate cell death in dose-dependent manners (Li et al., 2010). Moreover, Ca^2+^ overload- and ROS-associated mitochondrial dysfunction contributes to δ-tocotrienol (extracted from Annatto seeds)-mediated cell death in melanoma cells (Raimondi et al., 2021). A novel antitumor compound optimized from natural saponin Albiziabioside A induces caspase-dependent apoptosis and ferroptosis through the mitochondrial pathway as a p53 activator (Wei et al., 2018). It significantly suppresses tumorigenesis without causing toxicity in normal organs *in vivo*.

As mentioned above, considerable preclinical studies have suggested that plant-derived products are promising therapeutic agents against a range of cancers. Natural products from plants usually possess multi-targeting modes of action, and in this section, we focus on their function of inducing mitochondria-mediated cell death. Tripchlorolide, a bioactive component purified from the traditional Chinese botanical drug *Tripterygium Wilfordii Hook.f*. (Celastraceae), activates the mitochondrion-mediated apoptotic pathway involving the degradation of Bcl-2, the translocation of Bax from the cytosol to mitochondria, and the release of cytochrome C (Ren et al., 2003). Flavokawain A, a chalcone from kava plant, can significantly lose mitochondrial membrane potential and release cytochrome C into the cytosol in invasive bladder cancer cells (Zi and Simoneau, 2005). Consistently, *in vitro* and *in vivo* experiments showed that flavokawain A induces mitochondria-mediated apoptosis in a Bax protein-dependent manner and suppresses tumor growth (Zi and Simoneau, 2005; Liu S. et al., 2022). Avocatin B, a lipid derived from avocado fruit, and clitocine, a natural nucleoside extracted from wild mushrooms, disrupt mitochondria to trigger apoptosis (Sun et al., 2014; Lee et al., 2015). Moreover, robustaflavone A, a new bioflavonoid from plants, induces ferroptosis *via* the mitochondrial pathway in breast cancer cells (Xie et al., 2021).

In addition to phytochemicals, natural products from microbial sources appear to be promising modulators of apoptosis. Deoxynivalenol is a toxic secondary metabolite produced by Fusarium and can cause mitochondrial damage indirectly (Hou et al., 2021). It induces mitochondria-mediated apoptosis through inhibiting mitochondrial biogenesis and mitochondrial electron transport chain activity, ATP production, and mitochondrial transcription and translation. Therefore, we propose that rational use of deoxynivalenol with various mitochondrial toxicity has more potential for the treatment of tumors. The novel derivative (SL1) of levan produced from Bacillus subtilis NRC1aza shows high selective cytotoxicity against HepG2 cells (Abdel-Fattah et al., 2012). In terms of mechanism, SL1 induces apoptosis *via* mitochondrial pathway, which is initiated by the impairment of mitochondrial membrane potential and then released cytochrome c, that in turn activated caspase cascade and induced cell death. Short-chain fatty acids (SCFAs), the major by-products of bacterial fermentation of undigested dietary fiber in the large intestine, were shown to induce mitochondrial function-dependent growth arrest and apoptosis of colonic carcinoma cells in 1998 (Heerdt et al., 1998). Intriguingly, Tang et al. (2011) demonstrate that SCFAs-triggered autophagy serves as an adaptive strategy for retarding mitochondria-mediated apoptosis in human colon cancer cells.

Regarding marine sources, marine-derived natural products displayed a wide range of bioactivities, such as antitumor, anti-inflammatory, and anti-infective (Ren X. et al., 2021). Catassi et al. (2006) demonstrated that marine-derived agents trigger mitochondrion-mediated apoptotic pathway in non-small cell lung cancer cells. In terms of mechanism, marine-derived agents facilitate apoptosis through dephosphorylation of BAD serine136, BAD dissociation, cytochrome c release, caspase-3 activation, and cleavage of vimentin (Catassi et al., 2006).

**FIGURE 6:**
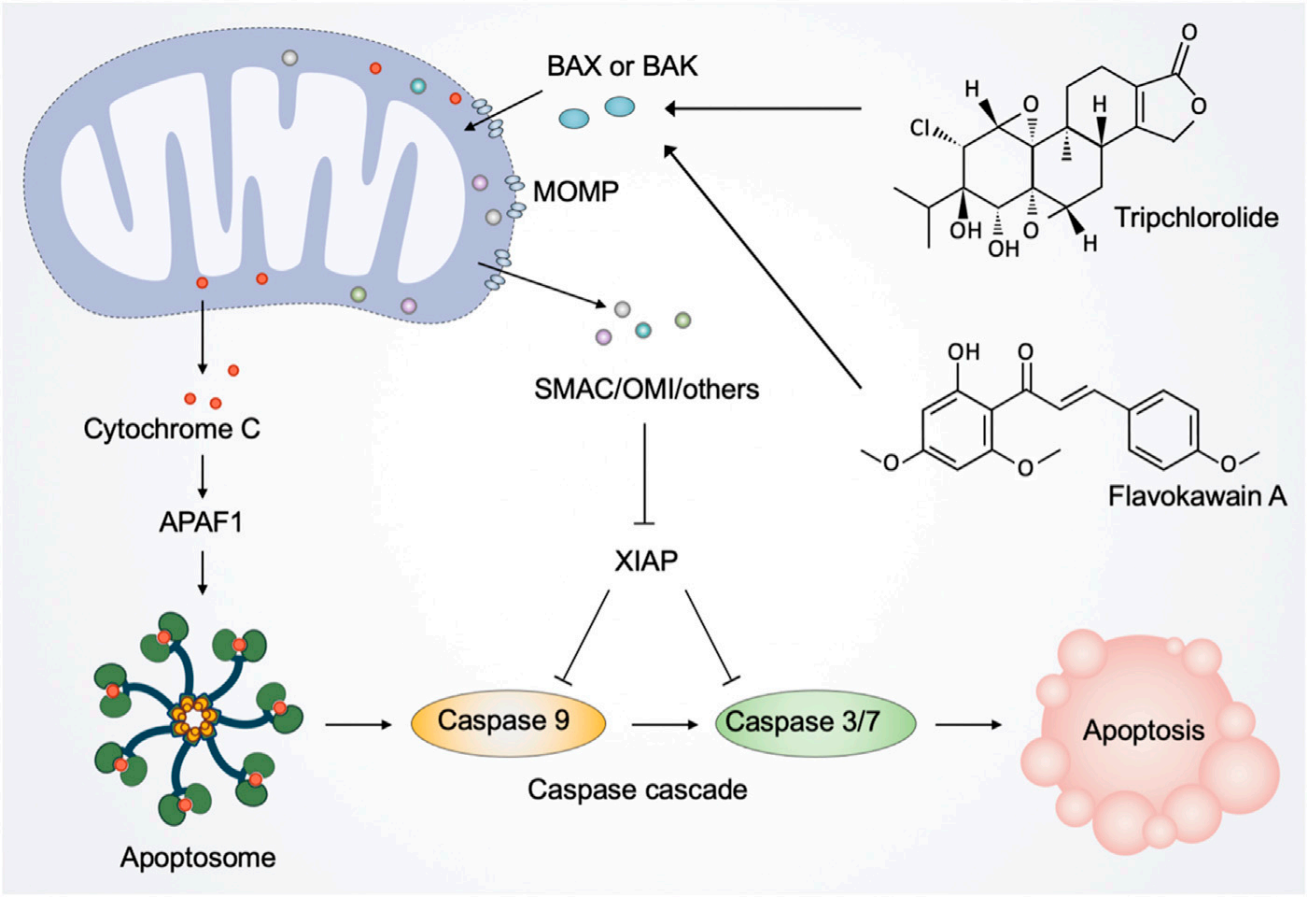
Natural products regulate mitochondria in apoptotic cell death. The activated pro-apoptotic effector, such as Bax and Bak, induce mitochondrial outer membrane permeabilization (MOMP). After the activation of MOMP, mitochondrial intermembrane space proteins, notably cytochrome C, are released into the cytosol. Once released, cytochrome C binds to the adaptor molecule APAF1 (apoptotic peptidase activating factor 1) to form a heptameric structure called apoptosome. Subsequently, apoptosome recruits and activates the initiator caspase 9, which in turn cleaves and activates caspase 3 and caspase 7, leading to the execution of apoptosis. Moreover, MOMP releases other proteins, including SMAC and OMI, to inhibit the caspase inhibitor XIAP (X-linked inhibitor-of-apoptosis protein), thereby facilitating apoptosis. Natural products such as tripchlorolide and flavokawain A promote the translocation of Bax from the cytosol to mitochondria and the release of cytochrome C into the cytosol, thereby activating the mitochondrion-mediated apoptotic pathway.

Taken together, many natural products from plants, microorganisms, and marine are cytotoxic and have shown their potential to eliminate different types of cancer cells *in vitro* and in animal models (Figure 6). Given their low toxicity and high effectiveness, natural products are considered direct sources of new chemotherapeutic agents to enhance efficacy or to ameliorate the side effects by regulating mitochondria.

## The future perspective and challenges

The recent development of pharmacology has provided new insights into drug discovery, especially focusing on natural products, offering the possibilities for regulating mitochondrial functions or mitigating mitochondrial dysfunction (Mu et al., 2020). As summarized in Table 1, many natural products have been reported over the past 10 years to be efficacious in modulating the mitochondrial quality control system and maintaining cellular homeostasis. In addition, many studies have shown that natural products can ameliorate mitochondria-originated ROS generation and reverse mitochondrial membrane potential disruption, thus protecting cells from oxidative damage (Madathil et al., 2012; Leirós et al., 2014; Alonso et al., 2016; Liang et al., 2021). These natural products hold promise for the development of novel therapeutic or preventive agents against diseases, including neurodegenerative diseases and diabetes (Liang et al., 2021). Interestingly, some natural products induce loss of MMPs, ATP depletion, Ca^2+^ overload, and generation of ROS, resulting in mitochondrial dysfunction and cell death (Yang et al., 2020). These natural products have shown antitumor efficacy against different types of tumors *in vitro* and *in vivo* through mitochondria-mediated cell death pathways. Accordingly, understanding the modulation function of natural products on mitochondria in depth may provide scientific insights for clinical applications of natural products. It is worth noting that several natural products with multi-target modes, as exemplified by the cases of resveratrol, baicalein, and EGCG, have intricate regulation on mitochondria, suggesting their potential use in diverse cellular effects (de Oliveira et al., 2015; Oliveira et al., 2016; Ashrafizadeh et al., 2020). There are challenges and opportunities in accurately determining dosing strategies to maximize the effect of natural products through single-agent or combination therapeutic strategies. Moreover, natural products frequently activate adaptive stress responses at low doses, whereas acute responses such as cell death are activated at high doses (Mattson and Cheng, 2006). Therefore, additional investigations are needed to identify patients most likely to benefit from different natural products and to determine the appropriate dose and duration.

**TABLE 1:** Natural products targeting mitochondrial dysfunction.

Natural products	Origin	Mitochondrial regulation	Experimental models	References
Epigallocatechin-3-gallate	*Camellia sinensis* L	Triggers mitochondrial biogenesis	*Caenorhabditis elegans* (during early-to-mid adulthood)	Xiong et al. (2018)
Resveratrol	*Vitis vinifera* L	Activates the PGC-1α/Sirt1/AMPK axis to trigger mitochondrial biogenesis	Down syndrome mouse model	Valenti et al. (2016)
Berberine	*Berberis vulgaris* L	Induces SIRT1-dependent mitochondrial biogenesis	Male Sprague Dawley rats (high fat diet)	Gomes et al. (2012)
Songorine	*Aconitum carmichaelii* Debx	Promotes cardiac mitochondrial biogenesis *via* Nrf2 and PGC-1α	C57BL/6 mice (with heart injury) and cardiac-specific Nrf2 knockdown mice	Li Y. et al. (2021)
Bouchardatine	*Bouchardatia neurococca*	Stimulates the SIRT1-LKB1-AMPK axis and triggers mitochondrial biogenesis	High-fat diet-fed mice	Rao et al. (2017)
Curcumin	*Curcuma longa* L	Improves MMP and ATP and restores mitochondrial fusion	The senescence-accelerated mouse-prone 8	Eckert et al. (2013)
Tetrahydrocurcumin	*Curcuma longa* L	Ameliorates homocysteine-mediated mitochondrial remodeling	Mouse brain endothelial cells (bEND3)	Vacek et al. (2018)
Quercetin	Fruits, vegetables, leaves and grains	Inhibits mitochondrial fission and mitochondrial enhances fusion	Hypobaric hypoxia-induced rats	Liu et al. (2015)
Hyperoside	*Abelmoschus manihot* L	Inhibited ischemia/reperfusion-induced mitochondrial fission	Renal ischemia-reperfusion injury mouse model	Wu et al. (2019)
Xanthohumol	*Humulus lupulus* L	Promotes mitochondrial fusion	Kainic acid-induced excitotoxicity rat model	Wang et al. (2020)
Liquiritigenin	*Glycyrrhiza radix*	Inhibits mitochondrial fragmentation	Neuroblastoma cells (SK-N-MC)	Jo et al. (2016)
Acacetin	Various plants (e.g., black locust)	Enhances mitophagy	d-galactose-induced cardiac senescence model	Hong et al. (2021)
Alliin	Garlic	Promotes PINK 1/Parkin-mediated mitophagy	THP-1 macrophages and mice (with lipopolysaccharide treatment)	Liu M. et al. (2022)
Polydatin	*Polygonum cuspidatum*	Mediates Parkin-dependent mitophagy	Acute respiratory distress syndrome mice model	Li et al. (2019)
Salvianolic acid B	*Salvia miltiorrhiza*	Suppresses the ROCK1-mediated mitophagy	Human umbilical vein endothelial cells (EA.hy926)	Ko et al. (2020)
Magnolol	Magnolia	Inhibit mitochondrial respiration	Oral cancer mouse model	Zhang et al. (2020)
Bergamot extracts	*Citrus bergamia* L	Inhibits the OXPHOS system	Human breast cancer cell lines (T47D and MCF7)	Fiorillo et al. (2018)
Baicalein	Scutellaria root	Promotes mitochondrial respiration	PC12 cells (with rotenone treatment) and male Sprague Dawley rats	Li et al. (2012)
Gastrodin	*Gastrodia elata*	Increases mitochondrial respiration	Human SH-SY5Y neuroblastoma cells (with H_2_O_2_ treatment)	de Oliveira et al. (2018)
Kaempferol	Various plants	Activates mitochondrial calcium uniporter to promote Ca^2+^ uptake	HeLa cell clone MM5 (expressing mitochondrially targeted mutated aequorin)	Montero et al. (2004)
Polydatin	Polygonum cuspidatum	Induces mitochondrial dysfunction-mediated calcium influx	Human colon cancer cells (parental and 5-FU-resistant HCT116 cells)	Bae et al. (2021a)
Biflavonoids	*Selaginella trichoclada*	Induces mitochondria-mediated ferroptosis	Breast cancer cells (MCF-7 cells)	Xie et al. (2021)
Levan derivative (SL1)	Bacillus subtilis NRC1aza	Induces mitochondria-mediated apoptosis	Human cell lines (HepG2, HCT-116, MCF-7, HeLa)	Abdel-Fattah et al. (2012)

Based on the structural classification of the natural products above, we found that the majority of natural products are phenolic compounds with aromatic ring and phenolic hydroxyl group. This is not surprising because phenolic compounds are well-known antioxidants and hold substantial ability to decrease oxidative damage (Rahman et al., 2021). Noticeably, the antioxidant activities of phenolic compounds are related to the substitution of hydroxyl groups in the aromatic rings of phenolics (Van Hung, 2016). Thus, analyzing and modifying the chemical structure of phenolic compounds may shed new light on the development of new drugs based on known structures and molecular groups.

As summarized in Table 1, the majority of mitochondria-targeted natural products are derived from plants, while only a few natural products are derived from animals and microbes, indicating the increasing potential of phytochemicals for pharmacological application. However, phytochemicals possess low bioavailability, poor pharmacological activity, and high metabolic decomposition rate, which hinder the transformation of plant-derived natural products from basic research to clinical application (Mahran et al., 2017; Takke and Shende, 2019). In addition, some natural products possess non-specific mitochondrial targeting properties, so it is of great significance to further develop structural modifications and derivatives on the basis of natural products. Inspiringly, due to the targeted and controlled drug release, nano-based drug delivery systems emerge as promising strategies to bring natural products to the forefront of drug development (Gunasekaran et al., 2014; Jha et al., 2022).

## Conclusion

In summary, increased research on mitochondria has facilitated the development of new strategies based on natural products to regulate mitochondrial dysfunction. Most of these natural products are phytochemistry, with minor amounts of metabolites derived from animals and microbes. As research progresses, it is believed that natural products with the ability to regulate mitochondria can offer new opportunities to treat various diseases. We hope that this review will provide new insights into the regulation of mitochondrial dysfunction by natural products and attract renewed interest in the application of natural products based on modulating mitochondria in the treatment of disease.

## Glossary

5-FU: 5-Fluorouracil
acetyl-CoA: acetyl coenzyme A
AD: Alzheimer’s disease
ADP: Adenosine diphosphate
AMP: Adenosine monophosphate
AMPK: Adenosine 5‘-monophosphate (AMP)-activated protein kinase
APAF1: Apoptotic peptidase activating factor 1
**ARE**: the antioxidant responsive element
ATP: Adenosine triphosphate
Cyt C: Cytochrome c
DRP1: Dynamin-related protein 1
EGCG: Epigallocatechin-3-gallate
ER: Endoplasmic reticulum
FAO: Fatty acid oxidation
FAD: Adenine dinucleotide
FDA: the Food and Drug Administration
FIS1: Fission protein 1
GPX4: Glutathione peroxidase 4
HCX: H^+^/Ca^2+^ exchanger
IMM: Inner mitochondrial membrane
LC3: Microtubule-associated protein light chain 3
LIR: LC3-interacting region
LKB1: Liver kinase B1
MCU: Mitochondrial calcium uniporter
MFF: Mitochondrial fission factor
MFN1: Mitofusins 1
MFN2: Mitofusins 2
MMP: Mitochondrial membrane potential
MOM: Mitochondrial outer membrane
MOMP: Mitochondrial outer membrane permeabilization
MRC: Mitochondrial respiratory chain
mtDNA: mitochondrial DNA
**NAD** ^**+**^: Nicotinamide adenine dinucleotide
NCLX: Na^+^/Ca^2+^/Li^+^ exchanger
NLRP3: NOD-like receptor thermal protein domain associated protein 3
NRF: Nuclear respiratory factors
OMM: Outer mitochondrial membrane
OPA1: Optic atrophy protein 1
OXPHOS: Oxidative phosphorylation
PD: Parkinson’s disease
PHD: Pyruvate dehydrogenase
PINK1: PTEN Induced Kinase 1
PGC-1α: Peroxisome proliferator-activated receptor gamma coactivator 1 alpha
PPARγ: Peroxisome proliferator-activated receptor- γ
PRX: Peroxiredoxins
ROCK1: Rho-associated protein kinase 1
ROS: Reactive oxygen species
SCFAs: Short-chain fatty acids
SIRT1: Sirtuin-1
STAT3: Signal transducer and activator of transcription 3
TCA: Tricarboxylic acid
TFAM: Mitochondrial transcription factor A
VDAC: Voltage-dependent anion channel
XIAP: X-linked inhibitor-of-apoptosis protein
