# Supplementary material for: The multifaceted roles of natural products in mitochondrial dysfunction
Source: Front Pharmacol. 2023 Feb 13;14:1093038. doi: 10.3389/fphar.2023.1093038 (PMC9968749; doi:10.3389/fphar.2023.1093038)
Supplement: Supplementary file 1 [file DataSheet1.DOCX]

Our analysis has been based on the use of several databases. We searched PubMed, ClinicalTrials, Google Scholar, and Web of Science to selected relevant studies published from inception to May,07,2022 without language restrictions.

The details are as follows:

The complete search used for Pubmed and Web of Science was ((Biological Products) OR (Products, Biological) OR (Biological Product) OR (Product, Biological) OR (Biologic Product) OR (Product, Biologic) OR (Biologic Products) OR (Biopharmaceuticals) OR (Biopharmaceutical) OR (Biological) OR (Biologic) OR (Biological Drug) OR (Drug, Biological) OR (Biologic Drugs) OR (Drugs, Biologic) OR (Biological Medicine) OR (Medicine, Biological) OR (Biological Medicines) OR (Medicines, Biological) OR (Biologicals) OR (Biologic Medicines) OR (Medicines, Biologic) OR (Biologic Pharmaceuticals) OR (Pharmaceuticals, Biologic) OR (Biologics) OR (Biologic Drug) OR (Drug, Biologic) OR (Biological Drugs) OR (Drugs, Biological) OR (Natural Products) OR (Natural Product) OR (Product, Natural)) AND ((Mitochondria) OR (Mitochondrion) OR (Mitochondrial Contraction) OR (Contraction, Mitochondrial) OR (Contractions, Mitochondrial) OR (Mitochondrial Contractions)).

In the ClinicalTrials, we used “Mitochondrial Dysfunction” as the term to search.

The complete search used for Google Scholar was ((natural products) OR (natural compounds)) AND ((mitochondria) OR (mitochondrial dysfunction)).
